# Electrical, structural, and autonomic atrial remodeling underlies atrial fibrillation in inflammatory atrial cardiomyopathy

**DOI:** 10.3389/fcvm.2022.1075358

**Published:** 2023-01-19

**Authors:** Yoshiko Murakata, Fumi Yamagami, Nobuyuki Murakoshi, DongZhu Xu, Zhonghu Song, Siqi Li, Yuta Okabe, Kazuhiro Aonuma, ZiXun Yuan, Haruka Mori, Kazutaka Aonuma, Kazuko Tajiri, Masaki Ieda

**Affiliations:** ^1^Department of Cardiology, Faculty of Medicine, University of Tsukuba, Tsukuba, Japan; ^2^Department of Cardiology, National Cancer Center Hospital East, Kashiwa, Japan

**Keywords:** experimental autoimmune myocarditis, EAM, neurotrophin, sympathetic nerve, myocarditis, inflammation

## Abstract

**Background:**

There is growing evidence indicating a close relationship between inflammation and atrial fibrillation (AF). Although underlying inflammatory atrial cardiomyopathy may contribute to the development of AF, the arrhythmogenic remodeling caused by atrial inflammation has not been elucidated in detail. Herein, we examined electrical, structural, and autonomic changes in the atria in a mouse model of autoimmune myocarditis.

**Methods:**

BALB/c mice were immunized with cardiac myosin peptide (MyHC-α_614–629_) conjugated with complete Freund’s adjuvant on days 0 and 7. Susceptibility to AF was assessed using right-atrial burst pacing.

**Results:**

The mice immunized with MyHC-α_614–629_ showed an inflammatory atrial cardiomyopathy phenotype, with enlarged atria; a high degree of inflammatory cell infiltration primarily consisting of CD4^+^ T cells, CD8^+^ T cells, Ly6G^low^CD11b^+^ macrophages, and CD11c^+^ dendritic cells; and severe interstitial fibrosis with collagen deposition. These mice demonstrated significantly enhanced susceptibility to AF, as indicated by their increased AF induction rate and duration. In addition, the expression of potassium channels (*Kcnh2, Kcnd3*, and *Kcnj2*) and calcium handling-associated genes (*Cacna1c*, *Camk2*, *Ryr2*, and *Atp2a2*) was downregulated. Connexin 40 expression was significantly downregulated, leading to frequent lateralization to the inflamed atrium. Sympathetic and parasympathetic innervation and neurotrophin expression (nerve growth factor and brain-derived neurotrophic factor) were upregulated in the inflamed atria.

**Conclusion:**

Inflammatory atrial cardiomyopathy promotes susceptibility to AF via arrhythmogenic electrical, structural, and autonomic remodeling of the atria.

## 1. Introduction

Atrial fibrillation (AF) is the most common clinically relevant arrhythmia, and is associated with significant morbidity and mortality (1, 2). Current treatment methods are suboptimal, and traditional anti-arrhythmic agents are relatively ineffective, necessitating more efficacious alternative therapeutic options for AF.

Mounting evidence indicates a close relationship between inflammation and AF (3, 4). The prevalence and prognosis of AF are associated with serum levels of inflammatory biomarkers, such as interleukin (IL)-6, C-reactive protein, tumor necrosis factor-α, and monocyte chemoattractant protein (MCP)-1 (5, 6). Lymphomononuclear infiltrates are commonly found in the right atrial biopsy specimens of patients with AF (7). Although underlying inflammatory atrial cardiomyopathy seen in AF may contribute to its development, the arrhythmogenic remodeling caused by atrial inflammation has not yet been elucidated in detail. Therefore, we aimed to examine the electrical, structural, and autonomic changes in the atria in a mouse model of autoimmune myocarditis.

## 2. Materials and methods

### 2.1. Animal experiments

Male BALB/c mice aged 7–9 weeks were purchased from CLEA, Japan, for use in the present study. All animal experiments were approved by the Institutional Animal Experiments Committee of the University of Tsukuba (ethics approval numbers: 20-039, 21-121 and 22-028) and were conducted humanely and in accordance with the Guide for the Care and Use of Laboratory Animals published by the US National Institutes of Health (NIH Publication No. 85-23, revised in 1996), the Regulation for Animal Experiments of our university, and the Fundamental Guidelines for Proper Conduct of Animal Experiments and Related Activities in Academic Research Institutions under the jurisdiction of the Ministry of Education, Culture, Sports, Science and Technology of Japan.

### 2.2. Immunization

The mice were immunized with 100 μg of murine cardiac α-myosin heavy chain (MyHC-α) peptide {MyHC-α_614–629_ [Ac-RSLKLMATLFSTYASADR-OH]; Toray Research Center, Tokyo, Japan} emulsified in 1:1 phosphate buffered saline (PBS)/complete Freund’s adjuvant (CFA) (1 mg/mL; H37Ra; Sigma-Aldrich, St. Louis, MO, USA) on days 0 and 7 (8–12).

### 2.3. Echocardiography

Transthoracic echocardiography was performed using the Vevo 2100 device (Fujifilm Visual Sonics), which is equipped with an MS-400 imaging transducer. Isoflurane induction was performed in an induction box with 3% isoflurane in pure medical oxygen. After the mouse’s righting reflex waned, it was fixed in the supine position on a heating pad to maintain normothermia, and then the electrocardiographic limb electrodes were placed. Anesthesia was maintained with 1% isoflurane treatment. The following echocardiographic parameters were measured: interventricular septum thickness at end-diastole, posterior wall thickness during diastole, left ventricular diameter at end-diastole, left ventricular diameter at end-systole, fractional shortening, ejection fraction, and left atrial volume.

### 2.4. Electrophysiological and AF induction studies

The protocols for the electrophysiological and AF induction studies have been described in our previous study (13). Briefly, right cervical vein cutdown was performed, and a 1-Fr quadripolar electrode catheter with an interelectrode distance of 2 mm (Unique Medical, Tokyo, Japan) was inserted into the right atrium for the electrophysiological study. The catheter was placed at a site where the amplitude of the intra-atrial electrogram was higher than that of the intraventricular electrogram. A programmable stimulator (SEN-7203; Nihon Kohden, Tokyo, Japan) was used to deliver approximately twice the threshold current for a duration of 2 ms (50 Hz) to measure the atrial effective refractory period (ERP). The ERP was measured at basic cycle lengths of 200 and 150 ms with a train of 8 basic stimuli (S1 × 8), followed by a single extrastimulus (S2) at 5-ms decrements. The ERP was defined as the longest S1–S2 interval in which capture failure occurred.

For the AF induction study, atrial burst pacing was delivered through two poles on the electrode catheter by a programmable stimulator with an amplitude of 5 V, cycle length of 20 ms, pulse duration of 6 ms, and stimulation time of 30 s. Each mouse was subjected to trial burst pacing for five consecutive repetitions to ensure AF induction. The AF induction rate was calculated by dividing the number of times AF was induced by the number of times burst pacing was performed. The duration of AF was defined as the interval from initiation to spontaneous AF termination. AF was only considered to have occurred when it lasted for more than 1 s.

### 2.5. Histopathological and immunohistochemical examination

The hearts were fixed in 4% paraformaldehyde in PBS and embedded in paraffin wax. For histological analysis, the sample tissue was cut into 3-μm-thick sections and stained with hematoxylin and eosin, and Masson’s trichrome. The proportion of the fibrotic area was calculated as the ratio of the connective tissue area (blue staining) to the total tissue area, excluding the blood vessels and the epicardial and endocardial planes, using ImageJ (version 1.53; National Institutes of Health, Bethesda, MD, USA). The area of fibrosis was quantified for three sections per atrium (6–8 fields per section).

Immunofluorescence staining was performed by incubating the sections in 10 mM citric acid buffer (RM102-C; LSI Medience, Tokyo, Japan) for antigen retrieval, followed by incubation with rabbit polyclonal anti-connexin 40 (anti-Cx40) antibody (ab101929; Abcam, Cambridge, UK), rabbit polyclonal anti-tyrosine hydroxylase (TH) antibody (AB152; Sigma-Aldrich, St. Louis, MO, USA), or rabbit polyclonal anti-choline transporter (ChT) antibody (ABN458; Sigma-Aldrich, St. Louis, MO, USA), with or without mouse monoclonal anti-α-actinin antibody (A7811; Sigma-Aldrich, St. Louis, MO, USA). Thereafter, the sections were incubated with Alexa Fluor^®^ 488-labeled goat anti-rabbit IgG (A11008; Thermo Fisher Scientific, Waltham, MA, USA) or Alexa Fluor^®^ 546-labeled goat anti-mouse IgG (A11030; Thermo Fisher Scientific, Waltham, MA, USA). The specimens were rinsed in PBS and mounted onto slides using a mounting medium containing 4’,6-diamidino-2-phenylindole. Subsequently, fluorescent images of the slides were captured using a digital fluorescence microscope (Biozero BZ-X700; Keyence, Osaka, Japan).

### 2.6. RNA extraction and quantitative reverse transcription polymerase chain reaction

All hearts that were resected for quantitative reverse transcription polymerase chain reaction (qRT-PCR) were snap-frozen and stored at –80°C. For preparation of total RNA, the tissue was homogenized using a bead kit (MagNA Lyser Green Beads; Roche Diagnostics, Indianapolis, IN, USA), according to the manufacturer’s instructions. Total RNA samples obtained from heart tissue were prepared using the RNeasy Fibrous Tissue Mini Kit (Qiagen, Hilden, Germany) and RNeasy Mini Kit (Qiagen, Hilden, Germany). Complementary DNA was synthesized from 1 μg of total RNA using the Omniscript RT Kit (Qiagen, Hilden, Germany). qRT-PCR analysis was performed using the LightCycler 480 System (Roche Applied Science, Indianapolis, IN, USA) with a Universal Probe Library (Roche Applied Science, Indianapolis, IN, USA). Hypoxanthine-guanine phosphoribosyl transferase (*Hprt*) RNA was used as the internal control. Gene expression values were calculated using the 2^–Δ^ ^Ct^ method.

### 2.7. Western blot analysis

Western blotting was performed as described previously (14). In brief, isolated hearts were homogenized in PRO-PREP™ protein extraction solution (iNtRON Biotechnology, Inc., Kyungki-Do, Republic of Korea) and the resulting supernatants were used for western immunoblotting. Appropriate volumes of the samples were mixed with an equal volume of sample buffer, heated at 95°C for 5 min, and then subjected to sodium dodecyl sulfate-polyacrylamide gel electrophoresis using 4–15% gradient polyacrylamide gels (Bio-Rad, Hercules, CA, USA). The proteins were transferred from the gels to polyvinylidene difluoride membranes using semidry electroblotting. Next, the blots were blocked and incubated with the following primary antibodies: signal transducer and activator of transcription 3 (STAT3) (ab119352; Cambridge, UK), phosphorylated STAT3 (phospho Y705) (ab76315; Cambridge, UK), and β-actin (sc-47778; Santa Cruz Biotechnology, Dallas, TX, USA). β-actin was used as the loading control because it is widely and consistently expressed in all types of eukaryotic cells, and its levels are known to remain stable during experimental treatments. The blots were incubated with an appropriate secondary antibody: horseradish peroxidase (HRP)-conjugated goat anti-rabbit IgG (ab6721; Cambridge, UK) or HRP-conjugated rabbit anti-mouse IgG (#7076; Cell Signaling, Danvers, MA, USA). The immunoreactions were visualized using the enhanced chemiluminescence method (ECL™ Prime Western Blotting Detection; GE Healthcare, Chicago, IL, USA). Densitometric analysis of the scanned immunoblot images was performed using ImageJ version 1.53 analysis software. The ratios of the densities of bands detected using phosphorylated antibodies to those detected using non-phosphorylated (total) antibodies were obtained from two independent measurements (n = 3 per group for each measurement).

### 2.8. Cytokine antibody array analysis

The hearts were homogenized in RPMI1640 containing 2.5% fetal bovine serum. Supernatants were collected after centrifugation and stored at –80°C. The concentrations of cytokines and chemokines in the heart homogenates were measured using semi-quantitative membrane-based cytokine array analysis (Mouse Cytokine Antibody Array; ab133993; Abcam, Cambridge, UK), according to the manufacturer’s instructions. Chemiluminescent detection was performed using ImageQuant LAS-4000 (GE Healthcare, Chicago, IL, USA) and densitometric data were obtained using ImageQuant TL software (GE Healthcare, Chicago, IL, USA). The mean intensities of the negative and positive control spots were used for background correction and normalization, respectively.

### 2.9. Flow cytometric analysis

Cardiac inflammatory cells were isolated and processed as previously described (15, 16). Flow cytometric analysis of the surface markers was performed by direct staining of the cell suspensions, which were isolated from the spleen and heart using fluorochrome-conjugated mouse-specific antibodies, followed by analysis with the FACSVerse instrument (BD Biosciences, San Jose, CA, USA). The following monoclonal antibodies were used for flow cytometric analysis: anti-CD3e (100306; Biolegend, San Diego, CA, USA), anti-CD4 60-0042-U100; TONBO Biosciences, San Diego, CA, USA), anti-CD8a (12-0081-81; eBioscience, San Diego, CA, USA), anti-CD11b (101227; Biolegend, San Diego, CA, USA), anti-CD11c (65-0114-U025; TONBO Biosciences, San Diego, CA, USA), anti-Ly6G (20-5931-U100; TONBO Biosciences, San Diego, CA, USA), and anti-CD45 (103114; Biolegend, San Diego, CA, USA).

### 2.10. Statistical analysis

All data were expressed as mean ± standard error of the mean. Normality was verified using the Shapiro–Wilk test. Statistical analyses were performed using the unpaired two-tailed *t*-test or Mann–Whitney *U* test for comparisons between two groups. Multiple comparisons were performed using the one-way analysis of variance with Tukey’s test or Kruskal–Wallis analysis with Dunn’s test. Results with *P*-values < 0.05 were considered statistically significant. All statistical analyses were conducted using GraphPad Prism software version 8.0 (GraphPad Software Inc., San Diego, CA, USA).

## 3. Results

### 3.1. MyHCα peptide immunization induces inflammatory atrial cardiomyopathy in mice

**FIGURE 1:**
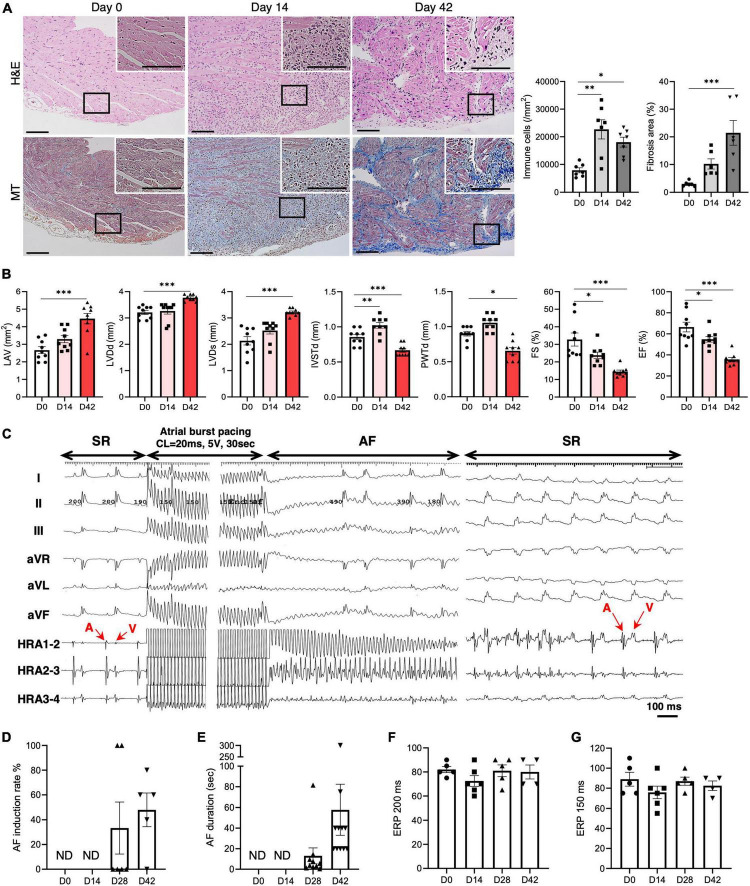
AF vulnerability in inflammatory atrial cardiomyopathy. **(A)** Representative histology of the atrium on days 0, 14, and 42 after immunization with MyHC-α (scale bars = 50 μm). The right bar graphs indicate the number of infiltrating mononuclear cells and fibrotic areas in the atria. **(B)** The bar graphs represent echocardiographic parameters at the indicated time-points. **(C)** The AF induction study was performed at baseline and 14, 28, and 42 days after MyHC-α immunization. Representative surface and endocardial electrocardiograms are shown. AF was induced by atrial burst pacing followed by spontaneous termination to sinus rhythm (SR). AF induction rate **(D)**, AF duration **(E)**, and right atrial ERP at basic cycle lengths of 200 ms **(F)** and 150 ms **(G)**. The results are presented as mean ± SEM. *P < 0.05, **P < 0.01, ***P < 0.001 using the Kruskal–Wallis test with Dunn’s test. MyHC-α, α-myosin heavy chain; AF, atrial fibrillation; ERP, effective refractory period; SEM, standard error of the mean.

The mice were immunized with MyHC-α on days 0 and 7. On day 14 (acute phase), a high degree of mononuclear cell infiltration was observed in the atrium and ventricle, which persisted until day 42 (chronic phase) (Figure 1A and Supplementary Figure 1). The degree of atrial fibrosis gradually increased during the study period (Figure 1A and Supplementary Figure 1). Echocardiographic examination revealed enlargement of the left atrium over time (Figure 1B). Ventricular dysfunction was also observed, as evidenced by an increased left ventricular diameter and decreased left ventricular contractility over time (Figure 1B). The left ventricular wall tended to be thicker in the acute phase, but was significantly thinner in the chronic phase.

### 3.2. AF vulnerability in inflammatory atrial cardiomyopathy

Subsequently, we examined the susceptibility to AF of this mouse model. An AF induction study was performed with rapid right atrium pacing at baseline and on days 14, 28, and 42 (Figure 1C). The mice demonstrated significantly enhanced susceptibility to AF, as indicated by the elevated AF induction rate and duration on days 28 and 42 (Figures 1D, E). However, the ERP did not change during the experimental period (Figures 1F, G).

### 3.3. Atrial inflammation and fibrosis in inflammatory atrial cardiomyopathy

**FIGURE 2:**
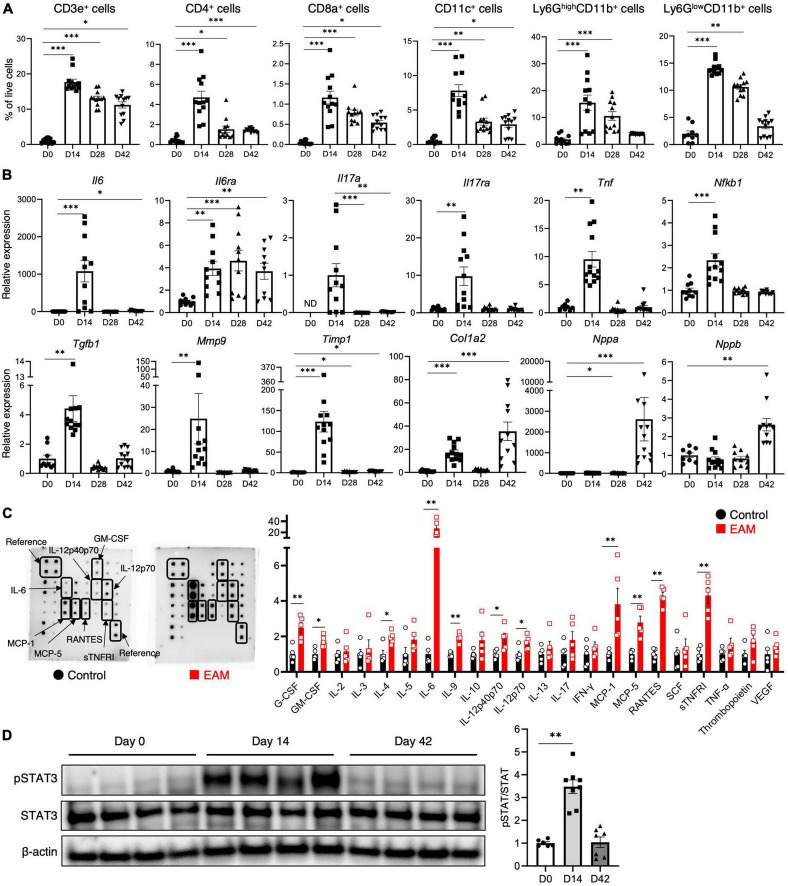
Inflammatory cell infiltration, cytokine expression, and STAT3 activation in inflammatory atrial cardiomyopathy. **(A)** Flow cytometric analyses of the cardiac-infiltrating cells in the atria. **(B)** Expression of inflammation- and fibrosis-related genes in the atria. **(C)** Semi-quantitative membrane-based cytokine array analysis of the atrium performed on day 14. The bar graph shows the signal intensity of each cytokine when the control was set to 1. **(D)** Western blot analysis of the atrium. The bar graph shows the densitometric ratio of phosphorylated STAT3 (pSTAT3) to total STAT3. The results are presented as the mean ± SEM. *P < 0.05, **P < 0.01, ***P < 0.001 using the Mann–Whitney *U* test, and Kruskal–Wallis test with Dunn’s test. STAT3, signal transducer and activator of transcription 3; SEM, standard error of the mean.

We performed an in-depth temporal analysis of the cellular infiltrate dynamics in the atria of the mice (Figure 2A). The infiltration of CD3^+^ T cells, CD4^+^ T cells, CD8^+^ T cells, CD11c^+^ dendritic cells, Ly6G^high^CD11b^+^ neutrophils, and Ly6G^low^CD11b^+^ monocytes/macrophages peaked on day 14, and was followed by a gradual decline on days 28 and 42. Supplementary Figure 2 depicts the temporal dynamics of cell composition of the spleen. As in the atria, the degree of inflammatory cell infiltration peaked on day 14 and declined to levels comparable to the controls during the chronic phase (day 42), except for neutrophil and monocyte/macrophage infiltration.

In the inflamed atria, the expression of most inflammation-related genes (*Il6*, *Il6ra*, *Il17a*, *Il17ra*, *Tnf*, and *Nfkb1*) was upregulated, especially in the acute phase (Figure 2B). However, the expression of *Il6a* decreased sharply on day 28, and *Il6ra* expression remained high until the chronic phase. Fibrosis-related genes (*Tgfb1*, *Mmp9*, *Timp1*, and *Col1a2*) were also upregulated in the atria, and collagen gene expression was particularly high in the chronic phase. In addition, the gene expression of the natriuretic peptides *Nppa* and *Nppb* was significantly elevated during the chronic phase. CFA used as an adjuvant had no effect on the expression of these genes (Supplementary Figure 3). Cytokine antibody array analysis was performed in the acute phase, and revealed higher expression of several inflammatory cytokines (granulocyte colony-stimulating factor, granulocyte macrophage colony-stimulating factor, IL-4, IL-6, IL-9, IL-12p40p70, IL-12p70, and soluble tumor necrosis factor receptor I) and chemokines [MCP-1, MCP-5, and regulated on activation, normally T-cell expressed and secreted (RANTES)] in the inflamed atria (Figure 2C).

IL-6 binds to the IL-6 receptor and triggers the Janus kinase associated with it, stimulating phosphorylation and activation of STAT3 (17). We found that STAT3 phosphorylation was significantly elevated in the atrium during the acute phase, and returned to baseline levels in the chronic phase (Figure 2D).

### 3.4. Electrical remodeling in inflammatory atrial cardiomyopathy

**FIGURE 3:**
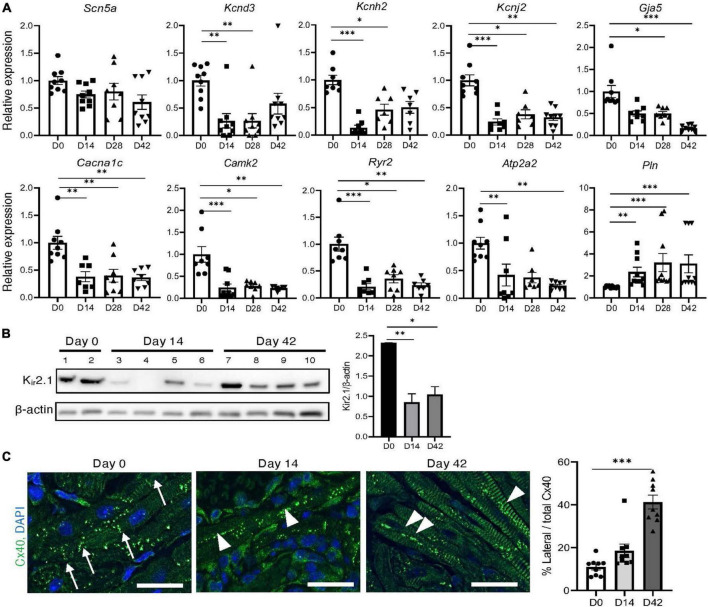
Electrical remodeling in inflammatory atrial cardiomyopathy. **(A)** Expression of electrical remodeling-related genes in the atria. **(B)** Western blot analysis of the atrium. The bar graph shows the ratio of Kir2.1 to β-actin. **(C)** Immunohistochemical analysis of Cx40 in the atrium. The arrows indicate organized Cx40 complexes at the junctional plaques between two adjacent cardiomyocytes. The arrowheads indicate Cx40 lateralization. Scale bars = 50 μm. The bar graph shows the Cx40 lateralization quantitative analysis results. *P < 0.05, **P < 0.01, ***P < 0.001 using the Kruskal–Wallis test with Dunn’s test. Cx40, anti-connexin 40.

Figure 3A illustrates the expression profiles of genes potentially involved in electrical remodeling of the atria. The expression of *Kcnh2* and *Kcnd3* was downregulated in the inflamed atria, particularly during the acute phase. K_ir_2.1 and its encoding gene *Kcnj2* were significantly downregulated during both the acute and chronic phases in the inflamed atria (Figure 3B). The expression of the calcium handling genes *Cacna1c*, *Camk2*, *Ryr2*, and *Atp2a2* was downregulated, while that of *Pln* was upregulated during both the acute and chronic phases of inflammation (Figure 3A). The cardiomyocyte surface localization of gap junction proteins influences the properties of cell-to-cell electrical conduction and wave front propagation (18). In the inflamed atria, expression of *Gja5* (the gene encoding Cx40) was significantly downregulated on days 28 and 42 (Figure 3A), and lateralization of Cx40 was frequently observed in the inflamed atria, particularly during the chronic phase (Figure 3C). CFA had no effect on the expression of electrical remodeling-related genes (Supplementary Figure 3).

### 3.5. Autonomic remodeling in inflammatory atrial cardiomyopathy

**FIGURE 4:**
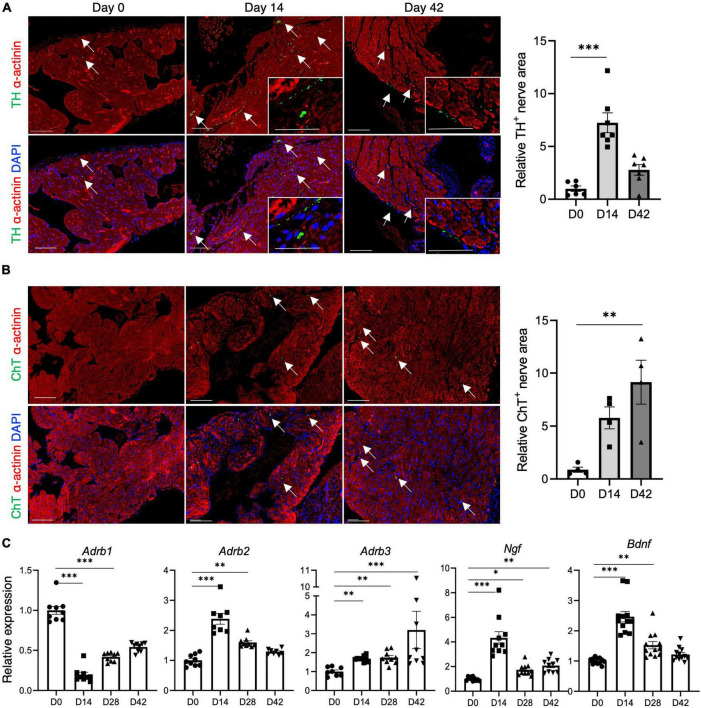
Autonomic remodeling in inflammatory atrial cardiomyopathy. **(A,B)** Immunostaining for TH **(A)** and ChT **(B)** in the atrium. Nerve areas (indicated by arrows) immunopositive for TH and ChT were determined using ImageJ software. The bar graphs show the relative TH^+^ or ChT^+^ nerve area when D0 was set to 1. Scale bars = 50 μm **(C)** qRT-PCR analysis of autonomic remodeling-related genes. *P < 0.05, **P < 0.01, ***P < 0.001 using the Kruskal–Wallis test with Dunn’s test. TH, anti-tyrosine hydroxylase; ChT, anti-choline transporter; qRT-PCR, quantitative reverse transcription polymerase chain reaction.

Immunostaining was conducted using antibodies to TH, a marker for sympathetic nerves, and ChT, a marker for parasympathetic nerves, to examine cardiac sympathetic and parasympathetic nerve innervation in inflammatory atrial cardiomyopathy. Sympathetic nerve innervation was upregulated in the acute phase (Figure 4A), while parasympathetic nerve innervation was upregulated in the chronic phase (Figure 4B). Neurotrophic factors, such as nerve growth factor (NGF) and brain-derived neurotrophic factor (BDNF), are involved in synaptogenesis and neurite outgrowth processes, supporting neuronal cell differentiation and maturation (19, 20). The gene expression of NGF and BDNF was upregulated in the inflamed atria, particularly during the acute phase (Figure 4C). The gene expression of β-adrenergic receptors differed according to the subtype. The gene expression of β1-adrenergic receptors decreased during the acute phase and recovered gradually during the chronic phase. Conversely, β2-adrenergic receptor expression increased during the acute phase and recovered gradually during the chronic phase, and β3-adrenergic receptor gene expression increased over time (Figure 4C). CFA had no effect on the expression of autonomic remodeling-related genes (Supplementary Figure 3).

## 4. Discussion

### 4.1. Main findings

In this brief research study, we characterized inflammation-induced structural, electrical, and autonomic remodeling of the atria, which potentially predisposes to the development of AF, using an experimental mouse model. The principal findings of this study were as follows: (i) mice immunized with MyHC-α_614–629_ exhibited the inflammatory atrial cardiomyopathy phenotype with atrial enlargement, a high degree of inflammatory cell infiltration, and severe interstitial fibrosis with collagen deposition; (ii) the mice demonstrated significantly enhanced susceptibility to AF, as indicated by the elevated AF induction rate and duration; (iii) the expressions of the potassium channels *Kcnh2, Kcnd3*, and *Kcnj2* and the calcium handling-associated genes *Cacna1c*, *Camk2*, *Ryr2*, and *Atp2a2* were downregulated; (iv) Cx40 expression was significantly downregulated, with frequent lateralization in the inflamed atria; and (v) sympathetic and parasympathetic innervation and neurotrophin expression were upregulated in the inflamed atria. These findings suggest that inflammatory atrial cardiomyopathy promotes AF vulnerability via electrical, structural, and autonomic remodeling of the atria.

### 4.2. Local inflammation-induced atrial remodeling

In this atrial inflammation model, AF was not induced during the acute phase (day 14), but during the chronic phase (day 28 onward) (Figures 2B, C), consistent with the findings of a previous study that utilized a rat model of experimental autoimmune myocarditis (21). However, changes in the properties of the atria, along with atrial inflammation, had already set in during the acute phase. Changes in the expression of potassium channels and calcium handling-related genes were already evident by day 14 (Figure 3A). The lateralization of Cx40 was observed frequently in the inflamed atria in the acute and chronic phases (Figure 3C). As shown in Figure 4, the onset of autonomic atrial changes also occurred during the acute phase. On the other hand, atrial fibrosis and dilation became prominent in the chronic phase (Figures 1A, B). Thus, local inflammation promotes electrical, structural, and autonomic remodeling of the atria and increases susceptibility to AF.

In our EAM model, atrial ERP was unchanged, consistent with previous studies in animal models of sterile pericarditis (22, 23). In these experimental pericarditis models, the increased atrial ectopy, which is associated with delayed afterdepolarization (DAD), and the triggered activity and atrial structural remodeling, which are characterized by myolysis and interstitial fibrosis, are associated with increased susceptibility to AF (24). On the other hand, in an angiotensin II (AngII)-injected mouse model that also induces atrial inflammation, AngII significantly decreased ERP (25). It also induced diastolic aberrant sarcoplasmic reticulum Ca^2+^ activities, prolonged action potential duration, and the appearance of DADs. Ang II also caused atrial interstitial fibrosis and left atrial dilatation which are associated with increased AF susceptibility. In EAM rats, AF was associated with a larger left atrium, cardiomyocyte hypertrophy, interstitial fibrosis, and reduced connexin 43 expression (21, 24). In our mouse EAM model, atrial fibrosis and atrial enlargement became increasingly pronounced from the acute to the chronic phase onward, and increased inducibility and duration of AF was also observed. Atrial fibrosis inhibits regional conduction and promotes maintenance of AF. Atrial regional conduction properties caused by interstitial fibrosis may favor the maintenance of AF in our EAM mice.

### 4.3. Autonomic remodeling in inflammatory atrial cardiomyopathy

Autonomic nervous system activation can induce significant and heterogeneous changes in atrial electrophysiology, and lead to AF (26). We found that sympathetic and parasympathetic innervation was upregulated, particularly in the acute and chronic phases, respectively (Figures 4A, B). The expression of NGF and BDNF was significantly upregulated (Figure 4C) in the inflamed atrium. NGF and BDNF are growth factors with important roles in synaptic and neuronal growth, myelination, differentiation, and neuronal survival (19, 20). Recent studies on depressive and cognitive disorders have revealed that inflammatory cytokines play a major role in the homeostasis of the nervous system and pathogenesis of neurological disorders (27–30). Microglia produce NGF and BDNF in response to IL-17A stimulation (28). IL-17A has a direct neurotrophic effect on sympathetic neurons and promotes sympathetic neurite outgrowth (30). IL-6 also plays a critical role in neural outgrowth, both directly and indirectly (27). Thus, atrial inflammation may result in autonomic imbalance, due to the direct or indirect effects of inflammatory cytokines, which can lead to AF susceptibility.

### 4.4. Potential limitations

First, the effect of the atrial load on AF associated with ventricular dysfunction cannot be disregarded, since our mice also developed ventricular myocarditis and reduced left ventricular contractility. Second, the mechanisms underlying the observed atrial remodeling were not investigated. Third, this study was performed in male mice only. Finally, as this study was conducted in mice, the degree to which our conclusions can be extrapolated to human AF development remains uncertain.

## Data availability statement

The original contributions presented in this study are included in the article/Supplementary material, further inquiries can be directed to the corresponding author.

## Ethics statement

The animal study was reviewed and approved by the Institutional Animal Experiments Committee of the University of Tsukuba.

## Author contributions

All authors listed have made substantial, direct, and intellectual contributions to the work, and approved it for publication.
