# Supplementary material for: Electrical, structural, and autonomic atrial remodeling underlies atrial fibrillation in inflammatory atrial cardiomyopathy
Source: Front Cardiovasc Med. 2023 Jan 19;9:1075358. doi: 10.3389/fcvm.2022.1075358 (PMC9892626; doi:10.3389/fcvm.2022.1075358)
Supplement: Supplementary file 1 [file Data_Sheet_1.PDF]

Supplementary Material

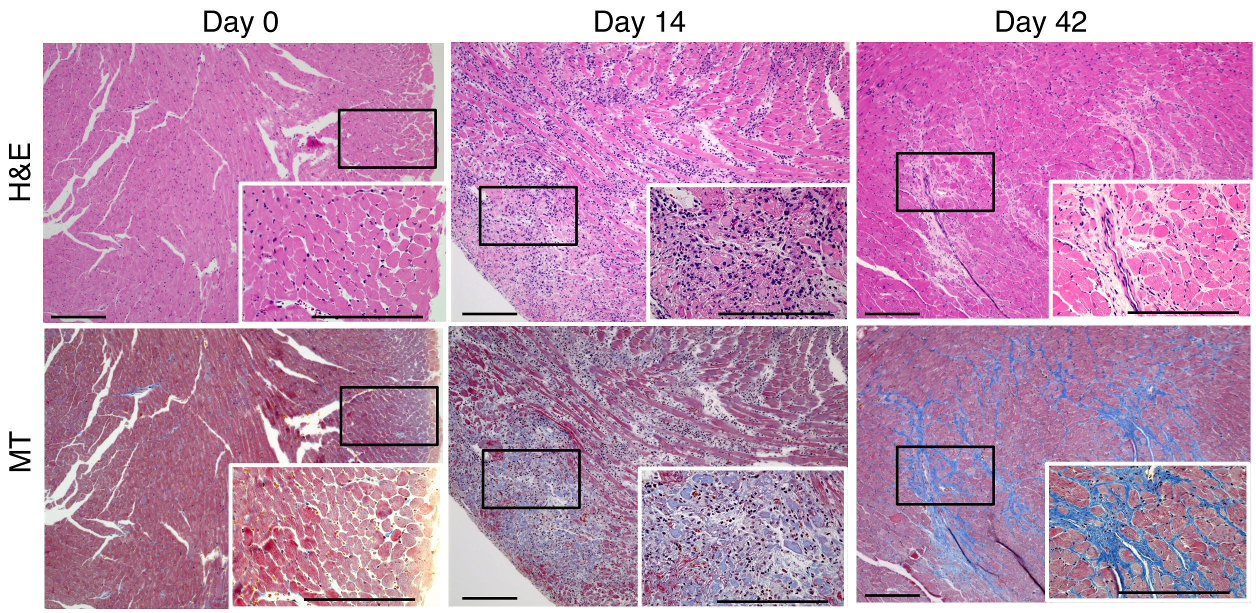

Supplementary Figure 1. Representative histology of the ventricle on days 0, 14, and 42 after immunization with MyHC-α. Scale bars = 50 µm. H&E, hematoxylin and eosin; MT, Masson's trichrome.

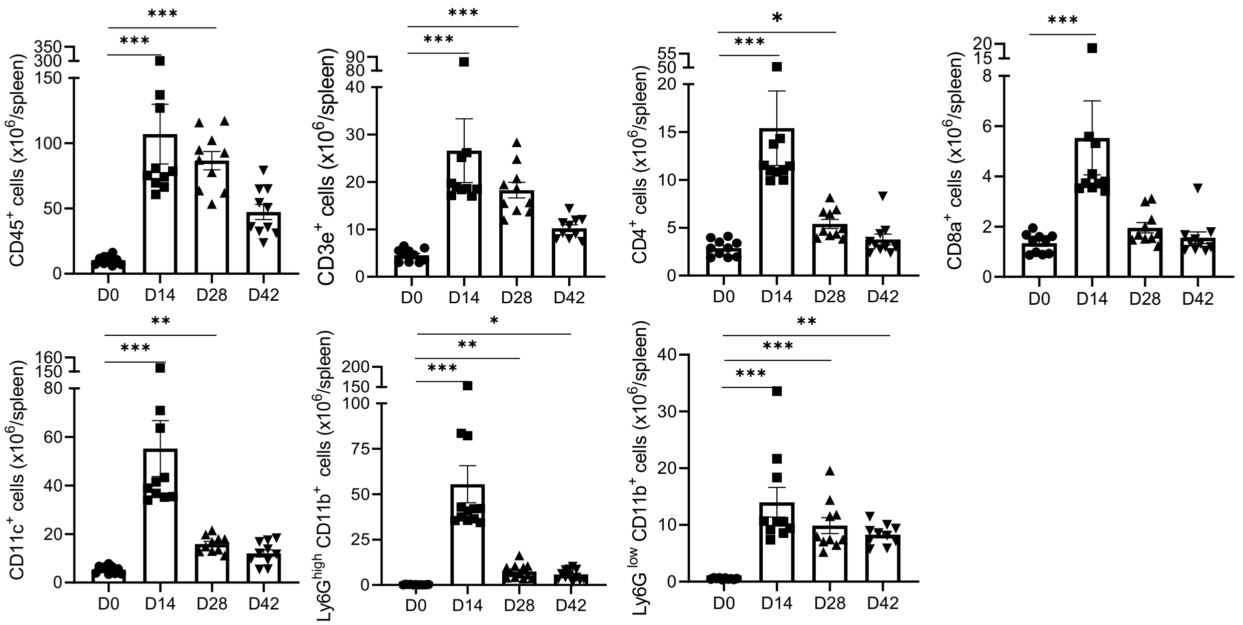

Supplementary Figure 2. Flow cytometric analyses of immunophenotypic profile of splenocytes. Results are presented as mean ± SEM. *$P < 0.05$, **$P < 0.01$, ***$P < 0.001$ by Kruskal–Wallis test with Dunn's test.

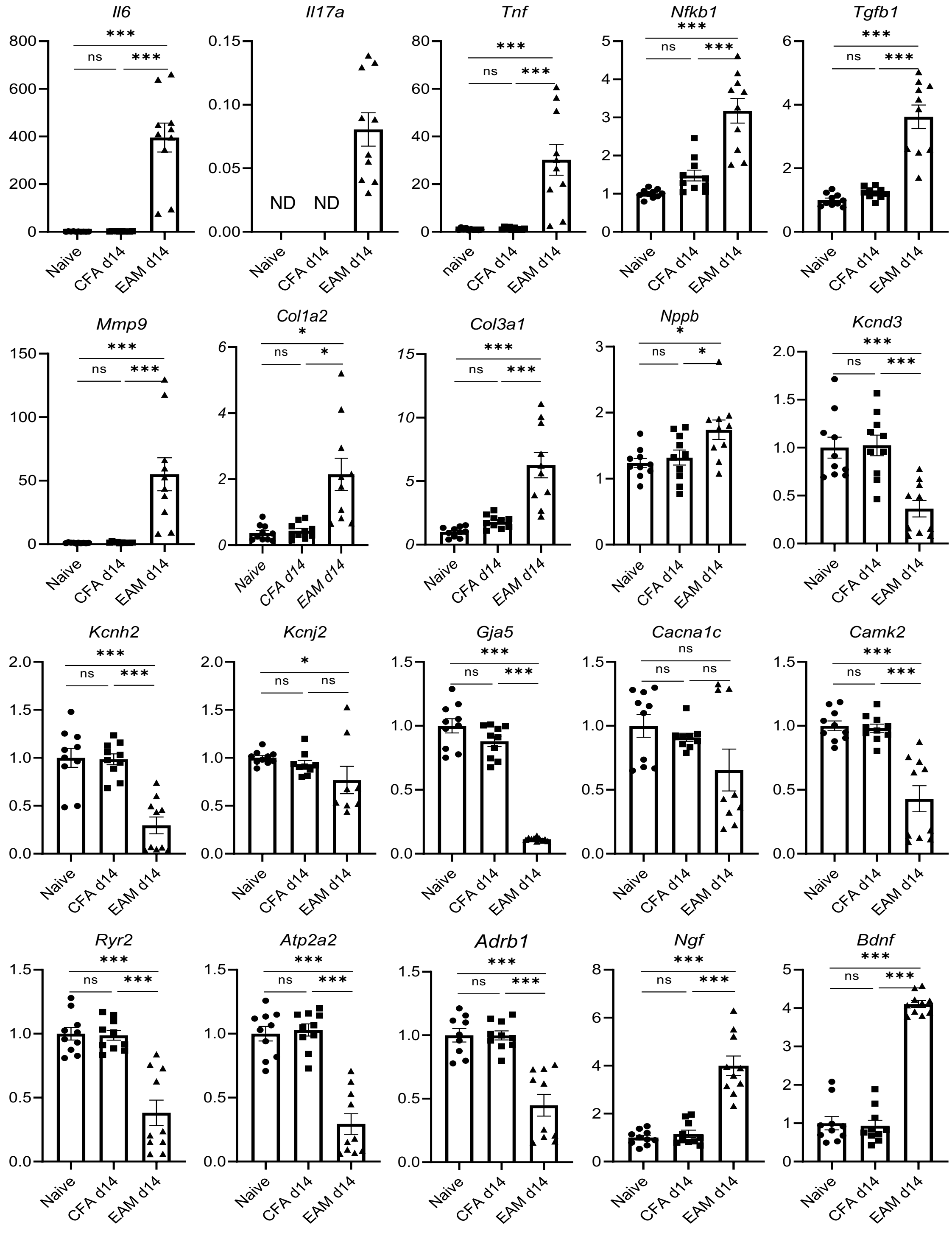

Supplementary Figure 3. Gene expressions in the atria. Mice were immunized with MyHC-α peptide emulsified in complete Freund's adjuvant (CFA), or with CFA alone on days 0 and 7. On day 14 (d14), the mice were sacrificed and their hearts were removed for qRT-PCR. We compared gene expressions among naïve (no immunization), CFA d14, and experimental autoimmune myocarditis (EAM) d14 (immunized with MyHC-α peptide emulsified in CFA). Results are presented as mean ± SEM. *$P < 0.05$, **$P < 0.01$, ***$P < 0.001$ by one-way analysis of variance with Tukey's multiple comparisons test.
